# Sclerosing angiomatoid nodular transformation of the spleen in a child with anemia: a case report and review of the literature

**DOI:** 10.1186/s13256-023-04144-0

**Published:** 2023-09-22

**Authors:** Neda Soleimani, Bita Geramizadeh, Mohammad Hossein Anbardar, Ali Bahador, Dornaz Safavi, Sahand Mohammadzadeh, S. Saeed Kasaee, Abbas Ayoub

**Affiliations:** 1 Department of Pathology, Shiraz Medical School, Shiraz University of Medical Sciences, Shiraz, Iran; 2 Department of Pathology, Abu Ali Sina Hospital, Shiraz Transplant Center, Shiraz University of Medical Sciences, Shiraz, Iran; 3 Shiraz Transplant Center, Abu Ali Sina Hospital, Shiraz University of Medical Sciences, Shiraz, Iran; 4 Department of Oncology and Hematology, Abu Ali Sina Hospital, Shiraz Transplant Center, Shiraz University of Medical Sciences, Shiraz, Iran; 5 Shiraz Transplant Center, Abu Ali Sina Hospital, Shiraz, Iran

**Keywords:** Child, Sclerosing angiomatoid nodular transformation, Spleen, Vascular neoplasm

## Abstract

**Background:**

Sclerosing angiomatoid nodular transformation of the spleen is a relatively rare benign vascular lesion in both adult and pediatric age groups with unclear etiopathogenesis and variable clinical presentations. Many benign and also malignant splenic masses could mimic sclerosing angiomatoid nodular transformation, both clinically and radiologically. Herein, we report our experience with a case of sclerosing angiomatoid nodular transformation in a 3-year-old girl.

**Case report:**

A 3-year-old Iranian girl presented with abdominal pain, back pain, and constipation for 2 weeks. She was being followed up by a pediatrician due to her short stature and persistent anemia. Physical examination showed stable vital signs, short stature, pallor, and a puffy face. Laboratory evaluation showed normochromic normocytic anemia with a normal reticulocyte count, ferritin, and hemoglobin electrophoresis. Radiologic assessments revealed a hypoechoic lesion in the spleen with high vascularity, clinically suspected to be lymphoma. She was operated on, and after partial splenectomy, pathologic evaluation of the spleen showed a solitary, well-demarcated, and unencapsulated dark mass. Microscopic examination revealed micronodular appearance composed of irregular-shaped vascular spaces lined by plump endothelial cells and surrounded by concentric collagen fibers, features in keeping with sclerosing angiomatoid nodular transformation. The patient’s anemia was resolved after surgery, and no clinical or radiologic deficits were noted during the 10-month follow-up visits.

**Conclusion:**

Although sclerosing angiomatoid nodular transformation is exceedingly rare in children, it should be considered a differential diagnosis in pediatric splenic neoplasms with concurrent hematologic manifestations, such as anemia.

## Introduction

Sclerosing angiomatoid nodular transformation (SANT) of the spleen is a relatively uncommon benign vascular lesion [1]. It is most common in the middle-aged population, with a female predominance; nevertheless, rare cases have been reported in children. The vast majority of patients have no symptoms, and most lesions are incidentally observed. However, a few cases might present with nonspecific symptoms, such as abdominal pain, nausea, vomiting, and malnutrition. Current imaging modalities provide a lot of preoperative information. However, some benign and malignant splenic tumors are still in the differential diagnosis of SANT, and the diagnosis is still a challenge without histological examinations [1–3]. Herein, we report a case of SANT in a child with unexplained anemia and clinical suspicion of lymphoma. Furthermore, we present a review of all English-language reported cases of SANT in the pediatric group.

## Case report

A 3-year-old Iranian girl presented with abdominal pain, back pain, and constipation for 2 weeks. She was born at term; nonetheless, she was under the follow-up of a pediatrician due to her short stature and persistent anemia observed 1 year prior. She had no fever, chills, night sweats, rash, arthralgia, myalgia, or lymphadenopathy. Other past medical and family histories were not significant. On physical examination, the vital signs were stable (blood pressure: 100/60 mmHg, pulse rate: 68 beats per minute, and temperature: 36.5 °C) with a 15 kg weight (75th percentile for age); however, she had short stature (85 cm, less than 5 percentile), pallor, and a puffy face. There was no abdominal tenderness or organomegaly.

**Fig. 1:**
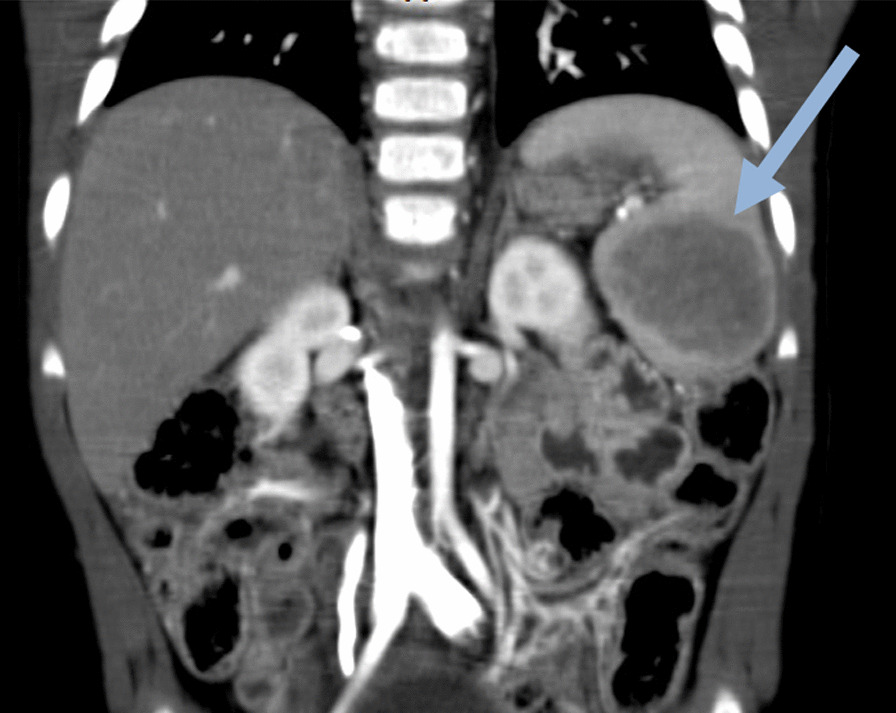
Computed tomography image of the patient shows an intrasplenic mass (arrow)

**Fig. 2:**
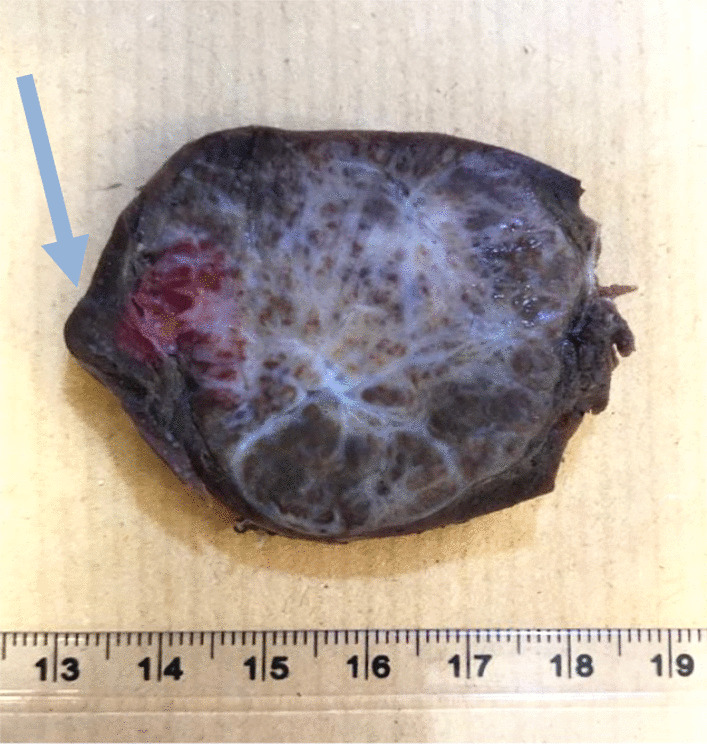
Cut section of spleen shows a firm, stellate, tan-white lesion with a thin rim of residual non-neoplastic spleen (arrow)

Laboratory evaluation showed anemia (hemoglobin: 8.4 g/dl) with a normochromic normocytic pattern (mean corpuscular volume: 84 fL, mean hemoglobin concentration: 27 pg), normal reticulocyte count (1%), ferritin, and hemoglobin electrophoresis. On radiologic evaluations, the spleen measured 92 mm × 70 mm and revealed a hypoechoic lesion (50 mm × 44 mm) with increased internal vascularity, which was in favor of lymphoma (Fig. 1). She underwent an elective exploratory laparotomy, partial splenectomy, and mesenteric lymphadenectomy. She was vaccinated against *Streptococcus pneumoniae* and *Neisseria meningitides* 2 weeks before surgery. The gross morphologic evaluation showed a solitary, well-demarcated, and unencapsulated dark mass (Fig. 2).

**Fig. 3:**
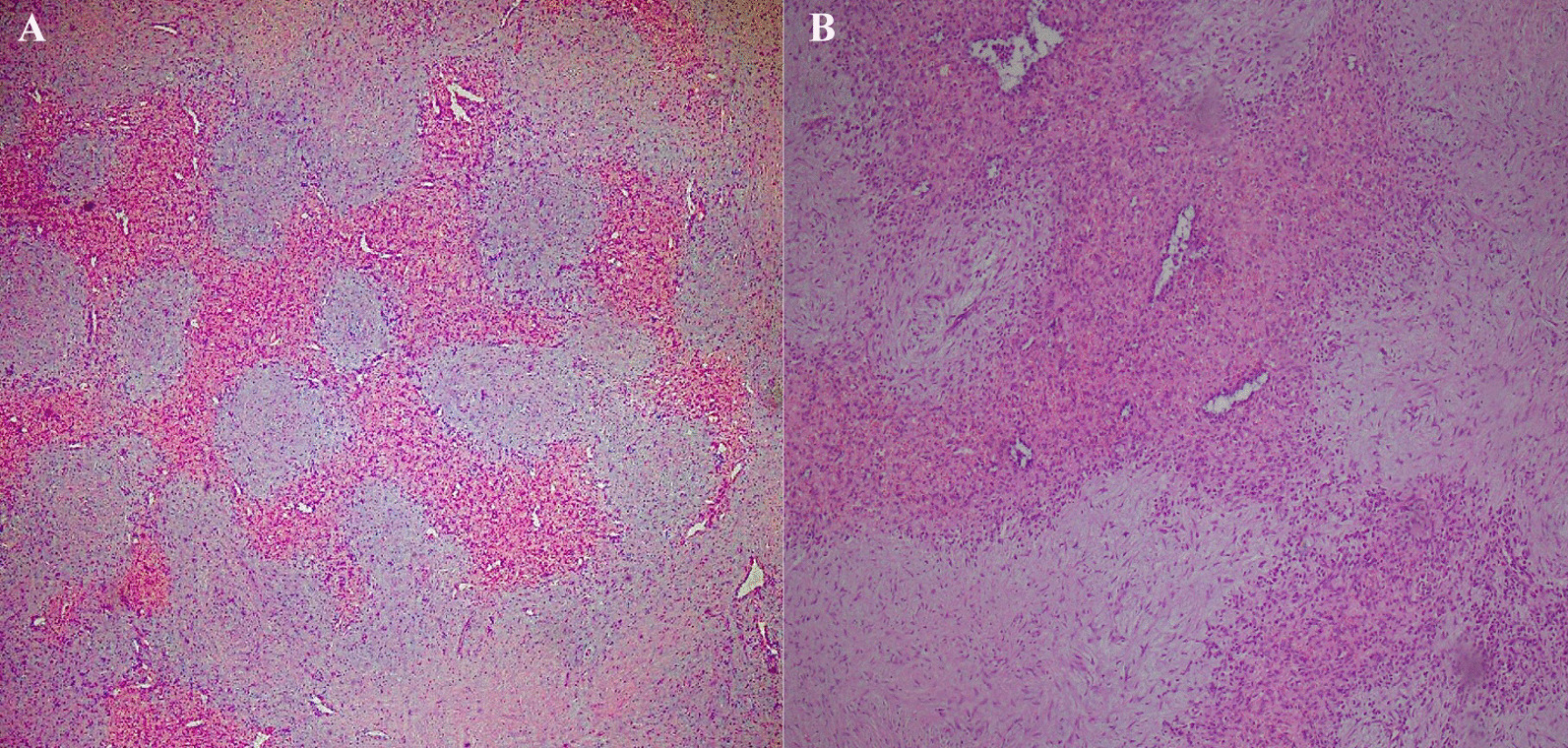
Low-power section of the resected mass shows multiple variably sized angiomatoid nodules within the red pulp separated by fibrosclerotic septa [hematoxylin and eosin (H&E) × 100] (**A**). The nodules are composed of well-formed capillaries arranged in a lobular pattern, sinusoid-like spaces, inflammatory cells, and histiocytes. There is no cellular atypia or necrosis (H&E × 200) (**B**)

**Fig. 4:**
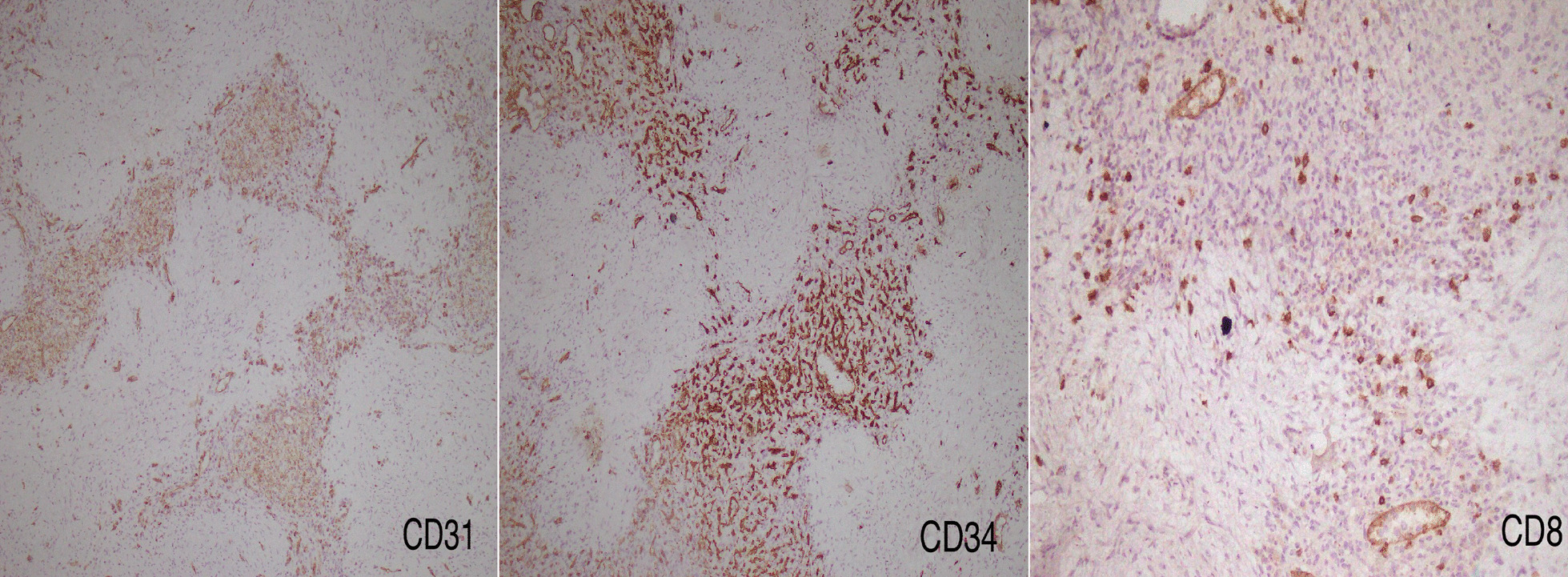
The immunostaining of the resected mass revealed three distinct kinds of vessels in the angiomatoid nodules positive for CD31, CD34, and CD8

Microscopic sections showed a micronodular appearance, composed of irregularly shaped vascular spaces lined by plump endothelial cells and surrounded by concentric collagen fibers, features in keeping with SANT (approved by immunohistochemistry) (Figs. 3, 4A–C). All the resected lymph nodes showed reactive changes. In a follow-up visit a month later, the patient showed improvement in her symptoms, and the anemia resolved (hemoglobin concentration: 12 g/dl). All workups for short stature (biochemical assessments and radiological tests for estimating bone age) showed appropriate results, and after receiving treatment for allergic sinusitis, the puffy face disappeared. Additionally, no clinical or radiologic deficits were noted during the 10-month follow-up visits. Informed consent was requested and obtained from the patient for publishing the case report and the accompanying images.

## Discussion

SANT is a rare vascular lesion of the spleen. It was previously labeled as a hamartoma of the spleen. However, in 2004, Martel *et al.* launched the SANT name based on an in-depth investigation of 25 cases. This new entity was described as a hamartoma-like lesion that arises from the red pulp and shows characteristic angiomatoid nodules. To date, only a few cases have been reported; however, the actual incidence and prevalence are still unknown. The SANT shows a marked female predominance (female to male ratio: 2.125). Most cases are incidentally observed in asymptomatic patients, although abdominal pain or discomfort, backache, and abnormal hematologic results occur in some cases [3–5].

Imaging findings are not specific and cannot usually distinguish between the various types of vascular lesions. However, typical computed tomography scan and magnetic resonance imaging findings include a solitary, round, and lobulated mass with early peripherally enhanced radiating lines, delayed enhancement of the fibrous tissue, and hypointense T2 signal intensity [6, 7]. Arising from the disorganized red pulp of the spleen, SANT has a distinct gross feature with a prominent multinodular appearance. Individual nodules have an ambiguously lobular architecture and are encircled by a hyaline shell. The vessels inside the nodules are cellular. The angiomatoid nodules consist of three different types of blood vessels with diverse immunohistochemical profiles, including capillaries (CD31+,  CD34+, and CD8−), small veins (CD31+, CD34−, and CD8−), and sinusoids (CD31+,  CD34−, and CD8+) [1, 8].

In contrast to its uncertain etiology and pathogenesis, SANT usually has a benign clinical course with no recurrence or complications. Most reported cases affected adult patients (less than 200 cases), with only a very small number of cases reported in the pediatric population [1, 9].

Many benign lesions, such as hemangiomas, hamartomas, littoral cell angiomas, and inflammatory myofibroblastic tumors, and some malignant lesions, such as low-grade lymphoma, are included in the differential diagnosis [6, 9, 10].

Hemangiomas are the most common benign primary neoplasm of the spleen and can show progressive enhancement, sometimes with a central fibrous scar; they may be distinguished from SANT by their high T2 signal intensity. Lymphoma is the most common malignant tumor of the spleen and can present as a solitary mass or multiple nodules; it has been described as isointense or hypointense relative to the spleen on T2-weighted images but typically shows little enhancement rather than a progressively enhancing pattern [6].

Many patients presenting with solid lesions in the spleen will eventually be diagnosed with a malignant tumor, but it is difficult to rule out the possibility of a malignant neoplasm preoperatively based on conventional imaging studies. So it is mandatory to make pathologic confirmation for the diagnosis and treatment of solid tumors [11].

SANT shows a distinctive nodular pattern, lack of atypia, and unique immunohistochemical profile in a core biopsy, but this procedure carries risks of bleeding and needle tract seeding. Therefore, splenectomy may be the preferred modality to rule out malignancy or other pathological processes [10–12].

In this report, we analyzed all the previously reported cases of pediatric SANT of the spleen in the literature (Table 1). Among these 14 cases, females and males were equally affected, in contrast to the female predominance pattern in adult cases; all SANT lesions were solitary, most cases were symptomatic, and among symptomatic cases, abdominal pain and discomfort were the most frequent symptoms. Moreover, about 50% of the cases had concurrent hematologic problems, such as anemia, thrombocytopenia, and an increased erythrocyte sedimentation rate. Anemia showed an iron deficiency pattern in most patients. Similarly, some previous reports had mentioned concurrent hematologic manifestations in splenic masses. In addition, anemia is a common feature in newly diagnosed patients with lymphoma, with anemia caused by chronic disease as the most common cause; however, our patient’s anemia was probably an anemia caused by chronic diseases, which resolved following mass resection [24].

**Table 1:** Demographic data, clinical data, and outcomes of pediatric cases with SANT

No.	Author	Age (years)	Sex	Clinical presentation	Laboratory data	Clinical and radiologic differential diagnosis	Size (GD) (cm)	Single versus multiple mass	Other remarkable findings	Outcome
1	Kuybulu *et al.* [13]	11	F	Incidentally found	Anemia Thrombocytopenia Increased ESR	–	11	Single	Short stature	Normal ESR
2	Bamboat *et al.* [14]	17	M	Intermittent abdominal pain	Leukocytosis Increased ESR	Atypical hemangioma /pseudotumor /Lymphoma	3.4	Single	–	No recurrence
3	Vyas *et al.* [15]	11	M	Flank pain	Within normal limits	–		Single	–	No recurrence
4	Agrawal *et al.* [16]	12	F	Abdominal discomfort	NA	–	NA	Single	–	No recurrence
5	Zhang *et al.* [17]	3	M	Incidentally found after car accident	Within normal limits	–	5	Single	–	No recurrence
6	Pelizzo *et al.* [18]	0.2	F	Abdominal distension and rectal bleeding	NA	–	NA	Single	–	NA
7	Delgado *et al.* [19]	4	M	Recurrent vomiting	Anemia Increased ESR Increased CRP	–	6	Single	Positive stool occult blood/ Esophagitis	NA
8	Cao *et al.* [5]	7	M	Incidentally found after trauma	NA	Hamartoma	9.5	Single	–	No recurrence
9	Idrissa *et al.* [20]	14	F	Incidentally found	Anemia Increased ESR Increased CRP	–	7	Single	urinary tract infection and chronic asthenia	No recurrence
10	Idrissa *et al.* [20]	4	M	Incidentally found	Within normal limits	–	3	Single	Cervical lymphadenopathy	No recurrence
11	Jamal *et al.* [21]	8	F	Recurrent epistaxis	Anemia Thrombocytopenia Increased PTT Increased reticulocyte count Decreased vitamin B12	Gaucher’s disease/ MPD/hemolytic disorders /hamartoma/ hemangioma /lymphangioma	NA	Single	Tachycardia, tachypnea	No recurrence
12	Sanmoto *et al.* [22]	14	F	Bleeding tendency Fatigue	Anemia Thrombocytopenia	Hemangioma/hamartoma/SANT	10.8	Single	–	No recurrence
13	Abboud [23]	16	M	Abdominal discomfort	Anemia Increased IgG	–	9.6	Single	–	No recurrence
14	Current case	3	F	Intermittent abdominal pain	Anemia	Lymphoma	5	Single	Short stature	No recurrence

*NA* not available, *GD* greatest diameter, *ESR* erythrocyte sedimentation rate, *CRP* c-reactive protein, *PTT* partial thromboplastin time, *MPD* myeloproliferative disorder, *SANT* sclerosing angiomatoid nodular transformation

## Conclusion

SANT is an uncommon splenic tumor. To our knowledge, 14 pediatric instances and less than 200 adult cases have been published in the English literature so far. SANT should be considered a differential diagnosis in pediatric splenic neoplasms with concurrent hematologic manifestations such as anemia, notwithstanding its rarity in children.

## Data Availability

All data generated or analyzed during this study are included in this published article.

## Abbreviation

SANT: Sclerosing angiomatoid nodular transformation
